# Audiovisual Processing of Chinese Characters Elicits Suppression and Congruency Effects in MEG

**DOI:** 10.3389/fnhum.2019.00018

**Published:** 2019-02-06

**Authors:** Weiyong Xu, Orsolya Beatrix Kolozsvári, Robert Oostenveld, Paavo Herman Tapio Leppänen, Jarmo Arvid Hämäläinen

**Affiliations:** ^1^Department of Psychology, University of Jyväskylä, Jyväskylä, Finland; ^2^Jyväskylä Centre for Interdisciplinary Brain Research, Department of Psychology, University of Jyväskylä, Jyväskylä, Finland; ^3^Donders Institute for Brain, Cognition and Behaviour, Radboud University, Nijmegen, Netherlands; ^4^NatMEG, Department of Clinical Neuroscience, Karolinska Institutet, Stockholm, Sweden

**Keywords:** audiovisual integration, magnetoencephalography, auditory cortex, language learning, reading, Chinese characters

## Abstract

Learning to associate written letters/characters with speech sounds is crucial for reading acquisition. Most previous studies have focused on audiovisual integration in alphabetic languages. Less is known about logographic languages such as Chinese characters, which map onto mostly syllable-based morphemes in the spoken language. Here we investigated how long-term exposure to native language affects the underlying neural mechanisms of audiovisual integration in a logographic language using magnetoencephalography (MEG). MEG sensor and source data from 12 adult native Chinese speakers and a control group of 13 adult Finnish speakers were analyzed for audiovisual suppression (bimodal responses vs. sum of unimodal responses) and congruency (bimodal incongruent responses vs. bimodal congruent responses) effects. The suppressive integration effect was found in the left angular and supramarginal gyri (205–365 ms), left inferior frontal and left temporal cortices (575–800 ms) in the Chinese group. The Finnish group showed a distinct suppression effect only in the right parietal and occipital cortices at a relatively early time window (285–460 ms). The congruency effect was only observed in the Chinese group in left inferior frontal and superior temporal cortex in a late time window (about 500–800 ms) probably related to modulatory feedback from multi-sensory regions and semantic processing. The audiovisual integration in a logographic language showed a clear resemblance to that in alphabetic languages in the left superior temporal cortex, but with activation specific to the logographic stimuli observed in the left inferior frontal cortex. The current MEG study indicated that learning of logographic languages has a large impact on the audiovisual integration of written characters with some distinct features compared to previous results on alphabetic languages.

## Introduction

Learning to read involves the integration of multisensory information (primarily from the auditory and visual modalities) and combining it with meaning. Multisensory integration, defined as modulation of brain responses by signals from multiple modalities, has been shown to be a dynamic and context-dependent process (van Atteveldt et al., 2014; Murray et al., 2016a). In natural audiovisual speech perception, it has been shown that complementary or correlated visual speech information could affect the auditory processing of speech by activating the perisylvian auditory speech regions through the ventral and dorsal visual streams (Campanella and Belin, 2007; Campbell, 2008; Bernstein and Liebenthal, 2014). Multisensory integration is also found within the visual cortices during early and late post-stimulus stages and could directly impact perception (Murray et al., 2016b; Kayser et al., 2017). Unlike spoken language, the ability to read is not hard-wired in the human brain through the evolution since written language is a recent cultural invention which has only existed for a few thousand years (Liberman, 1992). Consequently, it takes years of repetition and practice to form the long-term memory representations of audiovisual language objects, which would enable fluent readers to successfully automatize the integration of language-related auditory and visual sensory information (Froyen et al., 2009). A growing body of neuroimaging research has examined the neurophysiological basis of the letter-speech sound integration mainly in transparent alphabetic languages (Raij et al., 2000; van Atteveldt et al., 2004, 2009; Froyen et al., 2009). Interestingly, research using less transparent language such as English has found both similarities in the audiovisual integration effect compared with transparent languages and orthography dependent differences in the processing of irregular mappings of letter-speech sound combinations (Holloway et al., 2015). Therefore, an intriguing open question is about the audiovisual integration in other kinds of languages, for example character-speech processing in logographic languages such as Chinese. Understanding of such character-speech integration in logographic languages may provide more insights into the universal and language-specific brain circuits underlying audiovisual integration in reading acquisition.

Audiovisual paradigms, which typically consisted of auditory only (A), visual only (V), audiovisual congruent (AVC) and audiovisual incongruent (AVI) stimuli, were widely used in investigating brain mechanisms of multisensory interactions between the auditory and visual modalities (Raij et al., 2000; Murray and Spierer, 2009; van Atteveldt et al., 2009). Within the structure of such experimental design, two main approaches could be derived and used as indications of audiovisual integration. The first approach is based on the additive model, which compares the audiovisual responses to the summations of the constituent unisensory responses [AV − (A + V)], and has been frequently used in electrophysiological studies on multisensory integration (Raij et al., 2000; Calvert and Thesen, 2004; Stein and Stanford, 2008; Sperdin et al., 2009). The additive model could be applied to almost any kind of audiovisual experimental design with arbitrary auditory and visual combinations. This approach is suitable to detect both supra-additive [AV > (A + V)] and sub-additive (hereon referred to as the suppression effect) [AV < (A + V)] modulations of unimodal activities in the sensory-specific cortices as well as to observe new processes specifically activated by the bimodal nature of the stimulus under the assumption that there is no common activity (such as target processing) presented in the auditory, visual and audiovisual conditions (Besle et al., 2004). Animal electrophysiological studies have shown both super- and sub-additive multisensory interactions in the superior colliculus neurons and the superior temporal sulcus (STS; Meredith, 2002; Schroeder and Foxe, 2002; Laurienti et al., 2005; Perrault et al., 2005; Kayser et al., 2008; Stein and Stanford, 2008). Electroencephalography (EEG)/magnetoencephalography (MEG) studies on humans have typically shown suppressive multisensory effects (Schröger and Widmann, 1998; Foxe et al., 2000; Raij et al., 2000; Fort et al., 2002; Lütkenhöner et al., 2002; Molholm et al., 2002; Teder-Sälejärvi et al., 2002; Jost et al., 2014; Xu et al., 2018). Such suppression effects could occur as early as 50–60 ms after the stimulus onset and these functionally coupled responses are localized within the primary visual and auditory cortices as well as the posterior STS (Cappe et al., 2010). Other research in humans has also demonstrated that these audiovisual suppression effects can be observed at late time windows and for both familiar and unfamiliar audiovisual stimuli (Raij et al., 2000; Jost et al., 2014; Xu et al., 2018).

The second approach is to study the audiovisual congruency effect (Jones and Callan, 2003; Ojanen et al., 2005; Hein et al., 2007; Rüsseler et al., 2018), which compares different brain responses to congruent and incongruent audiovisual pairs. The rationale is that the congruency effect can only be established when the unisensory inputs have been integrated successfully (van Atteveldt et al., 2007a,b). One advantage of the congruency comparison is that it is not sensitive to any additional non-sensory activity and thus has a clear statistical criterion. Research using transparent languages such as Dutch and Finnish has found that the congruent alphabetic audiovisual stimuli elicit a stronger brain response in the superior temporal cortex than the incongruent pairs (Raij et al., 2000; van Atteveldt et al., 2004). Based on earlier studies (Raij et al., 2000; Besle et al., 2004; Cappe et al., 2010; Jost et al., 2014), the suppression effect reflects general audiovisual integration (including both early and late audiovisual interaction effects), whereas the congruency effect is more related to the specific interaction of learned or meaningful audiovisual associations (Hocking and Price, 2009).

Previous research has mainly focused on the brain mechanisms of audiovisual integration in alphabetic languages such as Dutch (van Atteveldt et al., 2004, 2009), English (Holloway et al., 2015) and Finnish (Raij et al., 2000). Several language-related and cross-modal brain regions have been shown to activate consistently during letter-speech sound integration in alphabetic languages. In particular, the superior temporal cortices have been reported in fMRI studies to have heteromodal properties (van Atteveldt et al., 2004, 2009; Blau et al., 2008). The left and right STS have also been implicated to be the main letter-speech sound integration regions in an early MEG study using Finnish letters (Raij et al., 2000). In addition, feedback projections from these cross-modal regions were found to alter the brain activities in the primary auditory cortex (van Atteveldt et al., 2004). The heteromodal areas in the temporal cortices have shown differences in their tolerances to temporal synchrony between modalities: the visual and auditory inputs are integrated in the STS and superior temporal gyrus (STG) within a broad range of temporal cross-modal synchrony between the auditory and visual stimuli, while the effect of congruent and incongruent auditory-visual stimuli rapidly diminishes with decreasing temporal synchrony in planum temporale (PT) and Heschl’s sulcus (HS) regions (van Atteveldt et al., 2007a). Top-down influences by different instructions and task demands also evidently affect the congruency effects (Andersen et al., 2004). For instance, different experimental designs (explicit/implicit and active/passive) have been shown to modulate the letter-speech sound congruency effect in fMRI (van Atteveldt et al., 2007b; Blau et al., 2008).

While fMRI provides accurate locations of the integration sites, its poor temporal resolution fails to capture the timing information. EEG and MEG have an excellent temporal resolution (in millisecond scale) and could provide additional timing information about audiovisual integration processes. An early MEG study using Finnish letters and speech sounds revealed that the auditory and visual sensory inputs showed maximal activities in multimodal sites about 225 ms after stimulus onset (Raij et al., 2000). It was followed by suppressive interaction at 280–345 ms in the right temporo-occipito-parietal junction and at 380–540 ms in the left STS and 450–535 ms in the right STS (Raij et al., 2000). Another MEG study using Hiragana graphemes and phonemes found that congruent audiovisual stimuli evoked larger 2–10 Hz oscillations in the left auditory cortex within the first 250 ms after stimulus onset and smaller 2–16 Hz oscillations in bilateral visual cortices between 250 and 500 ms compared with incongruent audiovisual stimuli (Herdman et al., 2006). Corroborating evidence comes from an EEG study that found audiovisual suppression effects in event-related potentials (ERPs) for familiar German (300–324 and 480–764 ms) and unfamiliar English words (324–384 and 416–756 ms), whereas audiovisual congruency effect could be found only for familiar German words (160–204, 544–576, 1032–1108 and 1164–1188 ms), and it was characterized by topographic differences probably due to lexical-semantic processes involved (Jost et al., 2014).

However, most studies on audiovisual integration have only investigated letter-speech sound mappings in alphabetic languages, and less is known about the logographic language such as Chinese. In Chinese, a single character encodes morphosyllabic information that represents a syllable with a distinct meaning (Perfetti and Tan, 1998; Shu, 2003; Tan et al., 2005; Ziegler and Goswami, 2005; McBride, 2015). For native Chinese speakers, long-term memory representations of characters through years of learning and reading repetition would enable them to retrieve the phonological and semantic information embedded in the characters directly. Semantic processing has been shown to modulate the brain activity around 400 ms, with the N400 response being sensitive to semantic incongruency (Kutas and Federmeier, 2011; Du et al., 2014; Jost et al., 2014). The magnetic counterpart of the N400 (N400m) has also been shown to be sensitive to semantic content and to have sources in the bilateral temporal cortices in unimodal conditions (Service et al., 2007; Vartiainen et al., 2009). Thus, research using logographic language such as Chinese is important to advance our understanding of the general mechanisms of audiovisual integration including cross-modal semantic congruency processing in the human brain.

In the current study, we investigated the dynamics of cortical activation to logographic multisensory stimuli using MEG. We designed an active one-back audiovisual cover task during which audiovisual integration could be examined. We presented Chinese characters and speech sounds as stimuli in A, V, AVC, and AVI conditions to native speakers of Chinese and used native speakers of Finnish, who were naive to Chinese, as a control group to verify the effects of long-term exposure to these stimuli. The MEG data were analyzed at both sensor and source levels to examine the suppression and congruency effects. Highly automatized audiovisual associations could affect processes related to general cross-modal integration manifested as suppression effect and congruency detection. More specifically, we hypothesized that learned characters combined with the corresponding congruent/incongruent speech sounds would elicit an audiovisual congruency effect manifested as a combined modulatory feedback and semantic N400m response, but only in native Chinese speakers. In addition, we examined the effects of long-term exposure of logographic language on the audiovisual suppressive interaction (sum of auditory and visual only conditions compared to audiovisual condition) which should be affected less by semantic processing than the congruency effect. Instead, suppressive integration is likely to reflect more general cross-modal processes. We thus expected more similar suppression than congruency effect to alphabetic languages found in earlier studies.

## Materials and Methods

### Participants

Chinese participants were adults and native speakers of Mandarin Chinese studying in Jyväskylä, Finland. Chinese participants had learned simplified Chinese characters through formal education. In addition, another group of native speakers of Finnish was recruited as a control group. Finnish participants had no prior exposure to the Chinese language. All participants included had normal hearing and normal or corrected-to-normal vision. They were screened for the following exclusion criteria: head injuries, ADHD, neurological diseases, medication affecting the central nervous system, language delays or any other language-related disorders. Ethical approval for this study was obtained from the Ethics Committee of the University of Jyväskylä. This study was carried out in accordance with the recommendations of the Ethics Committee of the University of Jyväskylä with written informed consent from all subjects. All subjects gave written informed consent in accordance with the Declaration of Helsinki. The protocol was approved by the Ethics Committee of the University of Jyväskylä. After the MEG experiments, all of them received movie tickets as compensations for their time. In total, we measured MEG data from 19 native Chinese speakers and 18 Finnish speakers. Of those seven were excluded from the Chinese group and five were excluded in the Finnish group for following reasons: four subjects due to poor visual acuity even with magnetically neutral glasses for vision correction, two subjects due to excessive head movements or low head positions in the MEG helmet, one due to technical problems during the recording, and five subjects due to strong noise interference and poor signal quality. The final dataset thus included 12 Chinese participants and 13 Finnish participants (Table 1).

**Table 1:** Demographic information of the study participants.

Group	Chinese	Finnish
Number of participants	12	13
Age	24.36 ± 3.66	24.31 ± 2.06
Sex	9 Female, 3 Male	9 Female, 4 Male
Handedness	12 right	12 right, 1 ambidextrous

### Stimuli and Task

The stimuli consisted of six Chinese characters (Simplified Chinese) and their corresponding flat tone speech sounds (1. 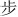: bu; 2. 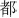: du; 3. 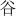: gu; 4. 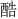: ku; 5. 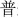: pu; 6. 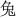: tu). The characters were all common characters familiar to the native Chinese speakers and had the following meanings: 1. steps/walking; 2. both/capital; 3. grain; 4. cool; 5. common; and 6. rabbit. The mean duration of the auditory stimuli was 447.2 ms (SD: 32.7 ms). The duration of the visual stimuli was 1,000 ms. Four kinds of stimuli, A, V, AVC and AVI were presented in random order with 108 trials in each type of the stimuli. Each trial started with a fixation cross at the center of the screen for 1,000 ms. In the AVI and AVC conditions, there was a 36 ms delay between the visual and auditory stimuli. This time delay is within the optimal range of cross-modal integration (Schroeder and Foxe, 2002; Kayser et al., 2008). The visual stimuli were projected onto the center of the screen one meter away from the participants with a white color background. The fixation cross was 1.2 cm and the characters were 2.5 × 2.5 cm on the screen. The stimuli were presented with Presentation (Neurobehavioral Systems, Inc., Albany, CA, USA) software running in a Microsoft Windows computer.

**Figure 1:**
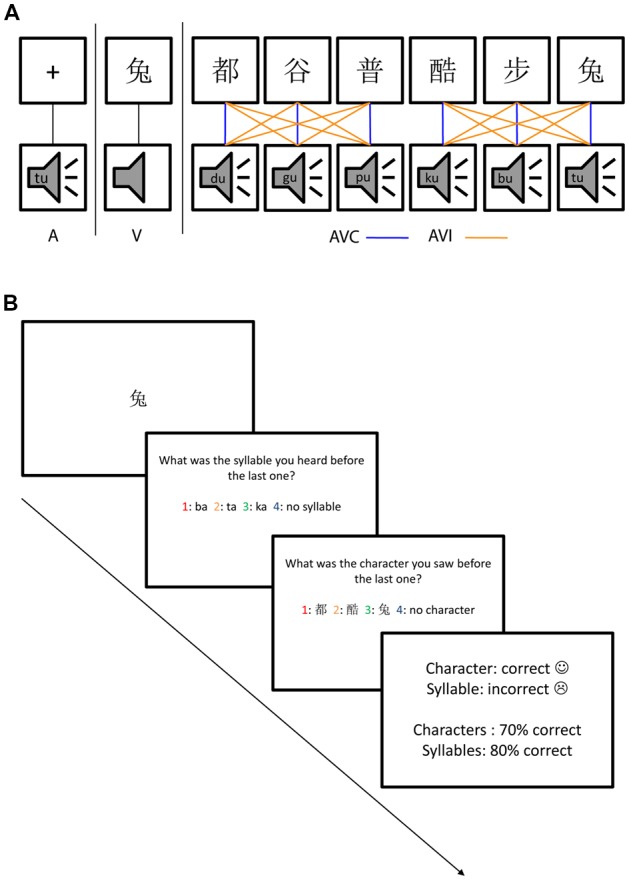
The audiovisual stimuli and dual one-back task. **(A)** Stimuli consisted of auditory only (A), visual only (V), audiovisual congruent (AVC) and AV incongruent (AVI) conditions using six Chinese characters and their corresponding speech sounds. The AVC combinations were indicated by blue lines and the incongruent combinations were indicated by yellow lines. The comparison between uni- and multimodal stimuli will reveal suppression effects while comparing the two multimodal conditions will reveal congruency effects. **(B)** The four types of stimuli (A, V, AVC, AVI) were presented randomly, as well as the cover task trials which occurred with 7.5% probability and consisted of one question for auditory, one for visual stimulus followed by feedback. The order of auditory and visual questions was also randomized.

Participants were instructed to do a dual-modality one-back task in order to keep their attention equally on both auditory and visual stimuli (Figure 1). Cover task trials as shown in Figure 1 occurred randomly (with 7.5% probability across all trials) to keep their attention on both auditory and visual modalities. The cover task trials tested the participant’s memory about the auditory and visual stimuli one trial before the last trial. The cover task trials consisted of one test trial for the auditory stimulus, one test trial for the visual stimulus followed by feedback. Four options were given on the screen in each of the auditory or visual test trials and the participants needed to choose the correct one from the four options using the response pad. The order of the auditory and visual test trials was randomized.

### MEG Recording

MEG data (102 magnetometer channels and 204 planar gradiometer channels; sampling rate: 1,000 Hz; band-pass filter: 0.1–330 Hz) were recorded using Elekta Neuromag^®^ TRIUX™ system (Elekta AB, Stockholm, Sweden) in a magnetically shielded room. The head position with respect to the MEG sensors in the helmet was monitored continuously with five digitized head position indicator (HPI) coils attached to the scalp. Three HPI coils were placed on the forehead and one behind each ear. At the beginning of each MEG recording, the positions of HPI coils were determined by three anatomic landmarks (nasion, left and right preauricular points), which were digitized using the Polhemus Isotrak digital tracker system (Polhemus, Colchester, VT, USA). To allow the co-registration with the MRI template, an additional set of points (>100) randomly distributed over the scalp were also digitized. Electro-oculogram (EOG) signal was recorded with one ground electrode attached to the collarbone and another two diagonally placed electrodes (one slightly above the right eye and one slightly below the left eye).

### Data Analysis

First, MEG data were processed with Maxfilter 3.0™ (Elekta AB, Stockholm, Sweden) to remove external magnetic disturbance and correct for head movements (Taulu et al., 2004; Taulu and Kajola, 2005; Taulu and Simola, 2006). Bad channels were marked manually and were excluded and later reconstructed in the Maxfilter. The temporal extension of the signal-space separation method (tSSS) was applied in buffers of 30 s (Taulu et al., 2004; Taulu and Kajola, 2005; Taulu and Simola, 2006). The head position was estimated using 200 ms time windows with 10 ms steps for head movement compensation. The mean head position across the whole MEG recording session was used for head position transformation.

MEG data were then analyzed using MNE Python (0.14; Gramfort et al., 2013). First, a low-pass filter of 40 Hz (firwin2 filter design, transition bandwidth 10 Hz) was applied to the continuous MEG recordings and data were segmented into epochs −200 to 1,000 ms relative to the stimulus onset. Data were then manually checked to excluded any trials that contaminated by head movement related artifacts or electronic jump artifacts. Then fast independent component analysis (ICA) was applied to remove any eye blink and cardiac artifacts related components. MEG epochs exceeding 1 pT/cm (for gradiometer channels) or 3pT (for magnetometer channels) peak-to-peak amplitudes were excluded from further analysis. Event-related fields (ERFs) were obtained by averaging trials in the four conditions (A, V, AVC, and AVI) separately. Sum of auditory and visual responses (A + V) was calculated by adding up the auditory and visual ERFs together (the numbers of trials in A and V conditions were equalized). To match the noise level of the A + V and AVC conditions and thus make these two conditions comparable, a subset of the AVC trials was created by randomly selecting about half the number of trials from the AVC condition as the number of trials in the A + V condition. For sensor level comparison, the gradiometer channel pairs in two orthogonal directions were combined using the vector sum method implemented in FieldTrip toolbox (Oostenveld et al., 2011). Gradiometers were chosen because they are less sensitive to noise sources originating far from the sensors than the magnetometers.

Individual magnetic resonance images (MRI) were not available from the participants and therefore the Freesurfer (RRID:SCR_001847) average brain (FSAverage) was used as a template for the source analysis. Three-parameter scaling was used to coregister FSAverage with the individual digitized head points. The average distance between the digitized head points and the scaled template scalp surface was 3.55 mm (0.25 mm SD).

Depth-weighted L2-minimum-norm estimate (wMNE; Hämäläinen and Ilmoniemi, 1994; Lin et al., 2006) was calculated for 10,242 free orientation current dipoles distribute on the cortical surface in each hemisphere. The noise covariance matrix was estimated from the raw MEG data from the 200 ms pre-stimulus baseline. The inverse solution was noise-normalized using dynamical statistical parametric maps (dSPM; Dale et al., 2000) for further statistical analysis. Since the FSAverage brain template that was used for all participants was only scaled to the subject-specific head size, the estimated brain activity could be directly compared in the statistical analyses without morphing to a common brain.

### Statistical Analysis

Sensor level statistical analyses on the combined gradiometers were conducted using the nonparametric permutation test (Maris and Oostenveld, 2007) with spatial-temporal clustering based on *t*-test statistic implemented in the FieldTrip toolbox. Source level analyses were similarly conducted using the nonparametric permutation *t*-test with spatio-temporal clustering in MNE Python. The time window was selected from 0 to 1,000 ms after the stimulus onset and the number of permutations was 1,024 in both sensor and source level statistical analyses. The cluster alpha was 0.05 for the incongruent–congruent comparison. For the AV − (A + V) comparison, a more conservative cluster alpha value of 0.005 was used due to the lower SNR since only half of the trials were averaged compared with the congruency contrast.

## Results

### Behavioral Performance

**Figure 2:**
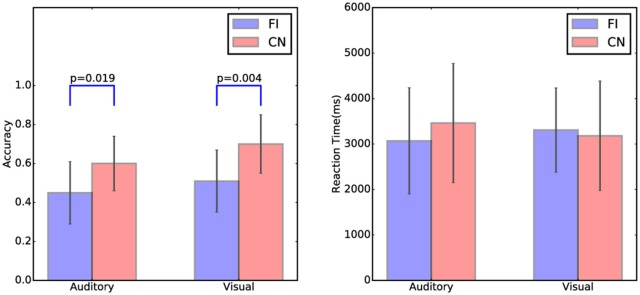
The accuracy and reaction time of the cover task trials in auditory and visual modalities and in the Chinese (*N* = 12) and Finnish (*N* = 13) groups. Error bars represent the standard deviation of the mean.

The accuracy and reaction time of the cover task trials in both Chinese and Finnish groups are shown in Figure 2. There was a significant group difference in the accuracy for both auditory (*t*_(23)_ = −2.52, *p* = 0.019) and visual modality (*t*_(23)_ = −3.20, *p* = 0.004) between Chinese and Finnish groups, with the Chinese participants (Auditory: mean = 0.60, SD = 0.13 ; Visual: mean = 0.70, SD = 0.15) being more accurate than the Finnish participants (Auditory: mean = 0.45, SD = 0.16; Visual: mean = 0.51, SD = 0.16) as expected. No significant differences in the reaction time were found between Chinese and Finnish groups.

### Grand Average

**Figure 3:**
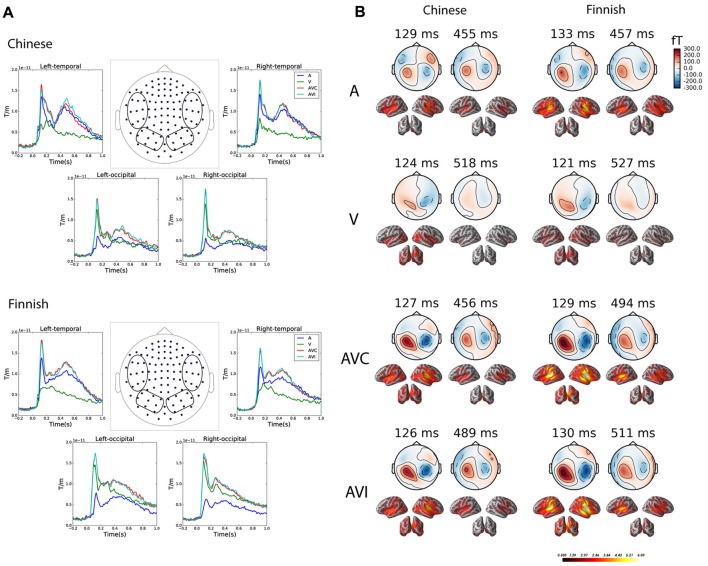
Grand average plots at both sensor and source level for the A, V, AVC and AVI conditions. **(A)** Grand averaged waveform for the combined gradiometer (vector sum of the paired orthogonal gradiometer channels) channels grouped (channels within the circle) over the left and right temporal and occipital channels in the Chinese (above, *N* = 12) and Finnish (below, *N* = 13) groups. **(B)** Magnetic field topography and dynamical statistical parametric maps (dSPM) source activation at the peak of grand average evoked responses in the early (100–200 ms) and late (300–700 ms) time windows for each of the four conditions.

Figure 3 gives an overview of the brain responses to the unimodal and bimodal audiovisual stimuli in sensor and source level in both Chinese and Finnish groups. Figure 3A shows the grand average evoked waveforms for the A, V, AVC and AVI conditions averaged over left and right temporal and occipital channels (vector sum of the paired orthogonal gradiometer channels) in the Chinese and Finnish groups. Figure 3B shows the magnetic field topography and dSPM source activations of the peak evoked responses at early (100–200 ms) and late (300–700 ms) time windows for each of the four conditions.

### Suppression Effect [AVC − (A + V)]

Testing for suppression effects in the latency range from 0 to 1,000 ms post-stimulus in both groups separately at the sensor level, the cluster-based permutation test revealed significant differences between the summed auditory and visual only conditions and the AVC conditions for both the Chinese group (*p* = 0.002) and the Finnish group (*p* = 0.006). In the Chinese group, the significant cluster was from 557 to 692 ms and mainly at the left temporal and frontal channels. In the Finnish group, the significant cluster was from 363 to 520 ms and mainly at the right parietal-occipital channels.

At the source level, the cluster-based permutation test also revealed significant differences between the summed auditory and visual only conditions and AVC conditions for both the Chinese group and the Finnish group. In the Chinese group, two significant clusters were found with different time windows and source locations. The first significant cluster was from 205 to 365 ms in the left angular and supramarginal gyri (*p* = 0.01). The second significant cluster was from 575 to 800 ms in the left temporal and frontal regions (*p* = 0.001). In the Finnish group, the significant cluster was from 285 to 460 ms and found in the right parietal-occipital regions (*p* = 0.003).

**Figure 4:**
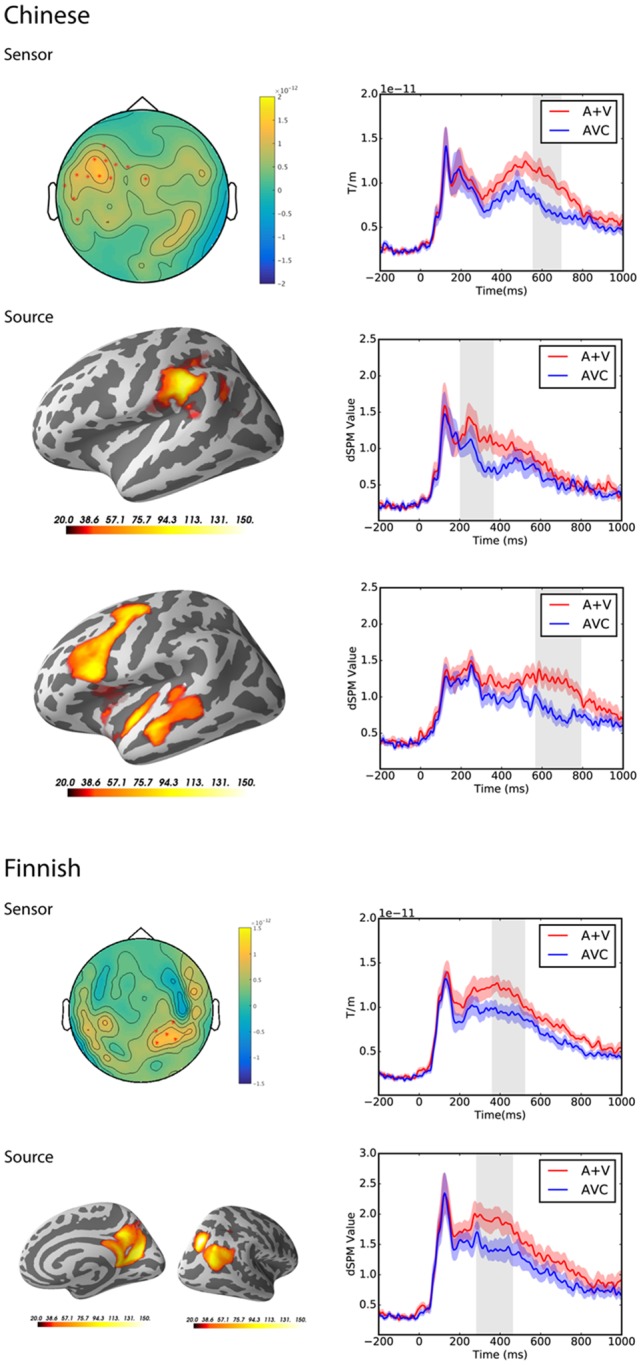
Sensor and source level statistical results for the suppression effects in the Chinese (*N* = 12) and Finnish (*N* = 13) groups. Left: significant clusters, represented by the red dots in the sensor space and the yellow and red coloring on the cortical surfaces for the source space. The brightness of the cluster was scaled by the temporal duration of the cluster in the source space. Right: average evoked responses from the channels of the significant cluster for the sensor space results, and the source waveform (dSPM value) extracted from the significant clusters for the source space results. The red and blue shaded area represents the standard error of the mean and the gray shaded area indicates the time window of the cluster.

Results for both sensor and source level suppression integration comparisons and the spatiotemporal pattern of significant clusters are shown in Figure 4.

### Congruency Effect (AVI − AVC)

**Figure 5:**
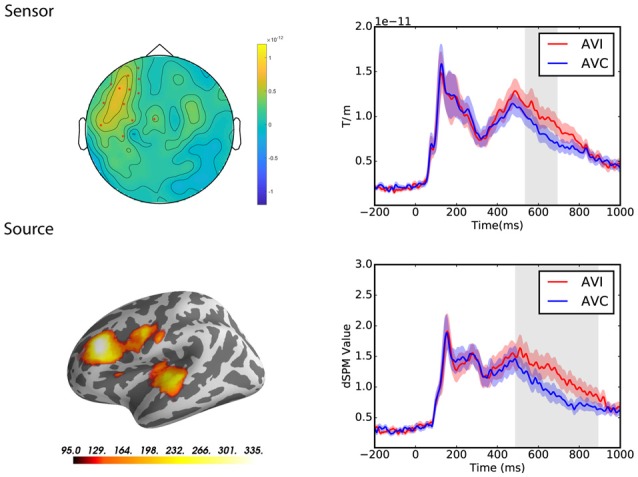
Sensor and source level statistical results of the congruency effects for the Chinese participants (*N* = 12). Left: the significant clusters, represented by red dots in the sensor space and the yellow and red coloring on the cortical surfaces for the source space. The brightness of the cluster was scaled by the duration of the cluster in the source space. Right: the average evoked responses from the channels of the significant cluster for the sensor space results, and the source waveform (dSPM value) extracted from the significant clusters for the source space results. The red and blue shaded area represents the standard error of the mean and the gray shaded area indicates the time window of the cluster.

At the sensor level, the congruency effect was tested in the latency range from 0 to 1,000 ms post-stimulus in both groups separately. The cluster-based permutation test revealed a significant difference between the congruent and incongruent conditions in the Chinese group only (*p* = 0.01; see Figure 5). The cluster occurred from 538 to 690 ms and the difference was most pronounced over the left frontal and temporal sensors in this latency range.

At the source level, the cluster-based permutation test also revealed a significant difference between the congruent and incongruent conditions in the Chinese group only (*p* = 0.008; see Figure 5). The cluster occurred from 490 to 890 ms and the difference was most pronounced over the left frontal and temporal regions.

## Discussion

In this study, we examined the effect of long-term exposure to Chinese characters on the audiovisual integration processes. We used a rigorous control of the participant’s attention on the two modalities by introducing a dual-modality one-back task. We observed that exposure to one’s native language and the long-term memory traces for spoken and written language indeed significantly changed the neural networks of audiovisual integration. This can be seen in the left-lateralized suppression effect, reflecting automatic audiovisual processes in the Chinese participants and a right-lateralized suppression effect in Finnish participants when processing novel Chinese audiovisual stimuli. Furthermore, long-term memory representations of Chinese characters are also manifested in how the brain reacts to the AVC and AVI stimuli in the left superior temporal and Broca’s area in native Chinese speakers.

Suppression (AVC vs. A + V) effects were found in both the Chinese and Finnish groups but with different hemispheric lateralizations and at different time windows. The suppression effect was most pronounced over the left angular/supramarginal, temporal and inferior frontal regions in the Chinese group, which suggested that the left hemisphere language network of native Chinese speakers was activated during processing of learned audiovisual association of Chinese characters and speech sounds. When children learn to read in an alphabetic language, visual letters and auditory phonemes are often presented simultaneously and neural pathways that map graphemes to phonemes are formed (Brem et al., 2010). Neural connectivity is strengthened through learning to read in school and later through reading practice, which would enable fluent readers to form long-term memory traces for written symbols and fast retrieval of audiovisual associations (Maurer et al., 2006). Consequently, the audiovisual suppression effect can be interpreted as the optimization of neural networks as a consequence of language learning in the Chinese participants (Raij et al., 2000). The early suppression effect was found in the left angular and supramarginal gyri about 200–350 ms after the stimulus onset. The left angular and supramarginal gyri have been considered as heteromodal areas related to linking orthographic representation from the occipital lobe to phonological coding represented in the STG (Price, 2000; Pugh et al., 2000; Schlaggar and McCandliss, 2007) and also possible feed forward connection to the inferior frontal gyrus (Simos et al., 2013). The left-lateralized suppression effect in the left superior and middle temporal areas in the later time window (about 550–800) matches those observed in earlier MEG, EEG and fMRI studies (Raij et al., 2000; Calvert et al., 2001; van Atteveldt et al., 2004) using alphabetic letters. The effect in Broca’s area is less often reported in audiovisual integration studies using alphabetic languages, possibly due to the fact that the Chinese language requires extra semantic processing in this area as shown by many other studies (Kuo et al., 2001; Tan et al., 2001; Wang et al., 2008). Taken together, it seems that the left superior temporal cortex is a common node in the neural network for audiovisual language processing for both alphabetic and logographic scripts. Importantly, the left inferior frontal cortex seems to be involved in the additional semantic-related audiovisual processing in logographic scripts (Wu et al., 2012; van Atteveldt and Ansari, 2014).

In the Finnish group, the suppression effect was most pronounced in the right inferior parietal and occipital area in a slightly earlier time window (285–460 ms), which partly matches with the findings by Raij et al. (2000) who also showed a predominant audiovisual interaction effect in the right temporo-occipito-parietal junction (280–345 ms). Clearly, for Finnish participants, Chinese characters did not have long-term memory representations and might be processed more like line drawings instead of meaningful characters. Parietal areas have been shown to be involved in multisensory processing in both human and monkey studies (Grunewald et al., 1999; Lewis et al., 2000; Bremmer et al., 2001). For example, lateral intraparietal area (LIP) which was originally considered as part of the unimodal visual cortex has been shown to respond to auditory stimuli in many studies (Bremmer et al., 2001; Ahveninen et al., 2006; Cohen, 2009). Other research has also indicated that the right parietal region plays a role in the perception of stimuli without any long-term representations arising simultaneously from the multiple sensory modalities (Kamke et al., 2012). The suppression effect in the right parietal and occipital regions at early time window indicates that the unfamiliar audiovisual information processing might rely more on the processing of the visual features (Calvert et al., 2001; Madec et al., 2016). It could be explained by the fact that Finnish participants who are naive to Chinese focused much more attention on the analysis of the spatial information of various strokes comprising the logographic character in order to be able to integrate the audiovisual stimuli. It should be noted that the suppression effect in the Finnish group was mostly related to this specific task and should not be interpreted as audiovisual integration related to logographic language. Therefore, the suppression effect found in the Finnish participants most likely represents a basic audiovisual processing (Calvert, 2001; Molholm et al., 2002; Cappe et al., 2010) of novel symbols in a working memory task.

Spatial-temporal clustering revealed an N400-like deflection elicited due to the congruency of the audiovisual stimuli in the Chinese, which was absent in the Finnish group. The congruency effect was mainly localized close to the left superior temporal cortex, Heschl’s gyrus, inferior frontal cortex and also parts of the insula. The difference occurred in a late time window around 500–800 ms. This result is in line with previous studies using MEG and EEG (Raij et al., 2000; Jost et al., 2014). According to the functional neuroanatomical model for the integration of letters and speech sounds proposed by van Atteveldt et al. (2009), the auditory and visual information is integrated in the heteromodal STS/STG and then feedback projected to the auditory cortex. In the current study, the congruency effect presented in the late time window therefore supports such feedback projection mechanism. Previous studies (Raij et al., 2000; van Atteveldt et al., 2004) using transparent alphabetic grapheme-phoneme associations found that the brain activations in the auditory association cortex were enhanced by congruent letters and suppressed by incongruent letters. This modulation is overruled during the explicit matching when both types of the audiovisual stimuli are equally relevant, independent of the congruency and temporal relation (van Atteveldt et al., 2007b). Whereas in opaque languages such as English, incongruent grapheme-phoneme associations have been shown to elicit a weaker and even reversed pattern of audiovisual integration to congruent pairs in the superior temporal areas (Holloway et al., 2015). Our results further indicated that audiovisual congruency is adaptive to different script types with a reversed direction in the superior temporal areas in logographic language compared to earlier reports on alphabetic language (Raij et al., 2000; van Atteveldt et al., 2004). Converging evidence from another audiovisual integration study (Jost et al., 2014) revealed a similar congruency effect for familiar German words, and was attributed to lexical-semantic processes involved in the processing of audiovisual words stimuli. The inferior frontal cortex has repeatedly been shown to be activated specifically by semantically incongruent audiovisual stimuli, which has been attributed to increased demands on the cognitive control involving semantic retrieval and working memory processes (Martin and Chao, 2001; Wagner et al., 2001; Doehrmann and Naumer, 2008).

The Chinese group showed a decrease of activation in the inferior frontal cortex when congruent audiovisual pairs were compared to the combined auditory and visual only stimulations. This indicates less neural resources for the audiovisual processing than the sum of resources it took to process the auditory and visual information independently. Furthermore, in the congruency comparison, the incongruent audiovisual pairs showed a higher activation than the congruent pairs again suggesting that it is less demanding to process the congruent audiovisual information due to complementary representations from both auditory and visual modalities. Since the Finnish participants had never learned the character-speech sound associations, they did not show any congruency effects. The left-lateralized suppression and congruency effects in the Chinese group suggest that the native Chinese could effectively utilize the audiovisual features of the learned language, whereas the right-lateralized suppression effect in the Finnish group may suggest that the Finnish participants rely more on the basic audiovisual processing mechanisms including substantial visual feature analysis. Therefore, congruency and suppression effects provide complementary information about audiovisual integration.

Both suppression and congruency effects in the Chinese group were mainly in the late time window about 500–800 ms after the stimulus onset. Previous studies have also found similar late orthographic-phonological interactions (400–700 ms) in spoken language processing during metaphonological tasks (Pattamadilok et al., 2011; Lafontaine et al., 2012). Interestingly, a late negativity enhancement (about 650 ms) after auditory stimulus onset was reported in mismatch negativity (MMN) studies on audiovisual integration in both the beginner and advanced readers in children (Froyen et al., 2009; Žarić et al., 2014, 2015). It was interpreted as more elaborate associative processes that are activated for the integration of letter-speech pairs in children. In addition, the suppression and congruency effects were mainly localized in the left frontal and superior and middle temporal areas. These locations are consistent with previous fMRI studies using Chinese characters and have shown that character reading process is characterized by particularly strong left lateralization of the frontal (BAs 9 and 47) and temporal cortices (Tan et al., 2000, 2001). For example, the left middle frontal area (BA 9) was suggested as an important region for integrating the logographic visuospatial analysis and the semantic processing in Chinese (Tan et al., 2001). Given the fact that the characters/speech items all refer to meaningful words, both the suppression as well as the congruency effect in the late time window most likely reflect a mixture of modulatory feedback and lexico-semantic processing during audiovisual integration in the Chinese participants. However, as discussed earlier, such audiovisual modulatory feedback and lexico-semantic processing seem to be largely overlapping in time windows and brain regions, further studies are needed to dissociate the effects of semantic processing from the other audiovisual process.

There are some limitations in the present study. We used an active paradigm which corresponded to real life situations of audiovisual integration (e.g., learning to read) as an active action. However, it also brought some challenges to identify the underlying functional brain networks while the participants were performing a working memory task. The brain activity during an active task makes the interpretation of direct group comparisons problematic since the task difficulties and demands for the two groups were different. Therefore, the conclusion regarding the orthographic influences on multisensory integration was indirectly based on the comparison of our results in the Chinese group with previous letter-speech sound integration studies in alphabetic languages. Another limitation relates to the lack of structural magnetic resonance images in the present study, which could potentially lead to poorer source localization accuracy. However, the localization of the brain activity at the millimeter scale was not the goal of the current study, instead we were interested in the estimation of the larger cortical areas that contributed to the cognitive process.

## Conclusion

Our findings demonstrated the effect of long-term exposure to logographic language on audiovisual integration processes for written characters and speech sounds. Different suppression effects in the Chinese and Finnish groups indicated the adaptive nature of the brain networks for processing different types of audiovisual information. Importantly, in the Chinese group, the left superior temporal and inferior frontal cortices were actively involved in the processing of both audiovisual vs. unimodal and congruency information pinpointing the left superior temporal and frontal cortex as important hubs for audiovisual and semantic processing in Chinese. The findings are remarkably similar to those found for alphabetic languages in the left superior temporal cortex but with also unique aspects of the processing of the logographic stimuli in the inferior frontal cortex. The results thus indicate that there are universal audio-visual association mechanisms in language learning complemented by language-specific processes.

## Author Contributions

WX, JH, and OK designed the study and performed the MEG experiments. WX, JH, and RO analyzed the data. All authors discussed the results and contributed to the final manuscript.

## Conflict of Interest Statement

The authors declare that the research was conducted in the absence of any commercial or financial relationships that could be construed as a potential conflict of interest.
